# Notice of Correction

**DOI:** 10.1080/22423982.2019.1589210

**Published:** 2019-04-02

**Article title**: Improving access to Indigenous medicine for patients in hospital-based settings: a challenge for health systems in northern Canada

**Authors**: Nicole Redversa, Justina Marianayagamb and Be’sha Blondina

**Journal**: *International Journal of Circumpolar Health*

**Bibliometrics**: Volume 78, Number 1

**DOI**: http://dx.doi.org/10.1080/22423982.2019.1577093

The above article was incorrectly published into the regular issue 1 of 2019. The article has been republished into the correct Special Issue: Collaborative approaches to wellness and health equity in the Circumpolar North: Proceedings of the 2017 Northern, Rural, and Remote Health conference with the DOI 10.1080/22423982.2019.1589208, Volume 78, Number 02.

